# Impact of Yoga on the Work-Life Balance of Working Women During COVID-19 Pandemic

**DOI:** 10.3389/fpsyg.2021.785009

**Published:** 2021-11-30

**Authors:** R. K. Roshni Raj Lakshmi, Elizabeth Oinam

**Affiliations:** ^1^Department of Yoga, Manipur University, Imphal, India; ^2^Faculty of Naturopathy and Yoga, Shree Guru Gobind Singh Tricentenary University, Gurgaon, India

**Keywords:** COVID-19, stress, work-life balance, gender inequality, yoga

## Introduction

COVID-19 has spread far and wide, and it has had a tremendous impact on all spheres of life. Infection with the virus and resultant illness and death has been seen all over the world. The pandemic has forced governments around the globe to take stringent measures for control of the spread. Quarantine, restrictions on traveling, reducing activities involving the gathering of crowds indoors, closing down of business set-ups, institutes, marketplaces have influenced one's daily lives drastically. Prolonged lockdown and restrictions have increased anxiety, depression, loneliness, and suicidal tendencies (Li et al., 2020).

## COVID-19 and Work-Life Balance

COVID-19 or Coronavirus disease is a form of Severe Acute Respiratory Syndrome that has spread rapidly worldwide since its outbreak in Wuhan, China. The infectious nature and high mortality rate of the disease have made it inevitable to take stringent measures to control the spread of the disease introducing many changes. The change has percolated down to work mode.

Work-life balance is “the division of one's time and focuses between working and family or leisure activities,” as defined by the Oxford English dictionary. Lockwood also defines work-life balance as “a state of equilibrium in which the demands of both a person's job and personal life are equal” (Lockwood, 2003). Inability to maintain the right work-life balance can have a devastating effect on one's health, personal relationships, and productivity at work *per se*. Maintaining a work-life balance even during regular times is a difficult task. During the pandemic, the new regulations in all sectors have made work-life balance even more elusive.

Remote working or work from home is the new mode of working. This causes a blurring of work time and family time as one is constantly at home. The burden of household responsibilities and work duties has increased during the lockdown. People in different sectors have been severely affected by the pandemic and resultant lockdown. People employed in medical set-ups are overloaded with work and facing more burnouts during the pandemic (Chen et al., 2021). Teaching faculties have moved from offline teaching to remote or online teaching. They are faced with new challenges of learning the ICT modes to facilitate teaching online. Besides a decrease in paper publication, academicians see a reduction in writing a research proposal and taking up leadership positions due to difficulty with work-life balance (Matulevicius et al., 2021). People employed in the real estate sector face a different set of challenges due to the pandemic. Lack of skilled laborers, difficulty in obtaining construction supplies, inability to complete projects on time, and absence of funds are some of the issues faced by the real estate sector during the pandemic (Majumder and Biswas, 2021).

Another important factor that aggravates the work-life balance is pre-school children, dependent or aged members of the family, and the unavailability of helpers in the pandemic situation. The constant presence of the individual at home makes them seem available for household chores and duties. On the other hand, remote working implies that the employee is constantly available and accessible to the employer round the clock (Lonska et al., 2021). There is no clear boundary between work time and personal time during the pandemic (Key Messages, 2020). Young children need to tending continuously, and working parents are overburdened without help from caretakers or crèche centers. Sick and aged members of the family also need extra care. All these factors lead to a chaotic situation and the increase in job loss, conversion of full-time employment to part-time employment, burnouts, and deterioration of familial relations during a pandemic. A support system should be available for the employees' welfare. When employees find support to achieve a positive work-life balance, they prove to be more motivated to perform their duty qualitatively.

## Gender and Work-Life Balance

Gender inequalities have always existed since time immemorial. Masculine and feminine types of work have always been well-demarcated. The concept of “work” place and “domestic” or “home” area was segregated with the industrial revolution when these two areas were markedly seen to be two very different things. The roles of a man and woman in a household were that of a “breadwinner” and “housewife”. Domestic chores and childcare were assumed to be the woman's responsibility, whereas the man had to be the one to go out and do a job to feed the family. Over time there were slight changes in the family dynamics, but as Connell puts it, it is only a “very good husband” who partially helps with the housework (Connell, 2005). Even with policies for working women to achieve work-life balance, women are responsible for adjusting and work out the family balance. Especially in a patriarchal society like India, the gender disparity is more profound, and women-home association is more potent. In the pandemic situation, gender inequality concerning work-life balance is much more deplorable. Even in countries like Iceland, which is known for reducing the gender gap, the gender norms can be exaggerated (Hjálmsdóttir and Bjarnadóttir, 2021). The situation can worsen in other countries, with women academicians reporting more conversion to part-time employment, reduction in paper publication, and writing up research proposals due to work-life conflict (Matulevicius et al., 2021). Chung et al. have noted that flexible work time is more convenient for women (Chung et al., 2021). But during the Covid-19 pandemic, women have faced disparities at work, with more women facing layoffs, furloughs, and pay cuts (Del Boca et al., 2020). COVID-19 has amplified the gendered pressures and women's labor force exit should be observed in context with the gendered burdening at workplace. Gender is a strong predictor of worse mental health in women predicting anxiety and depression (Docka-Filipek and Stone, 2021). The prerequisites of maintaining work-life balance is a stable and balanced mental state. Also, disruption in work-life balance implies amplified stress. Yoga is a mind-body medicine modality that has beneficial neuro-physiological impact on stress and improves mental health.

## Yoga and Work-Life Balance

Yoga is a science that teaches the ideal way of life, drawing its origin from ancient India. It focuses on bringing one's existence to a balanced state. It is a system of mind-body medicine that has a salubrious effect on all aspects of being. It is a complementary and integrative healthcare practice that focuses on bottom-up neurophysiological and top-down neurocognitive mechanisms to enhance psychological well-being (Sullivan et al., 2018). Bharathi and Mala have found that yoga and meditation are personal enhancers that help women working in the IT sector achieve work-life balance (Bharathi and Mala, 2016). It is also found that yoga and meditation help reduce stress in employees complaining of burnout (de Bruin et al., 2017). Intensive care nurses have a busy and stressful jobs, often putting them at risk of burnout. A study has shown that yoga and meditation can be effective strengtheners of well-being in intensive care nurses (Jarden et al., 2019). Sharma et al. have demonstrated that online yoga could be a viable mechanism to improve mental and physical health for employees during the lockdown (Sharma et al., 2020). Another study has shown that online yoga can help reduce perceived stress, depression, mental well-being, and coping skills in people working from home during the pandemic (Wadhen and Cartwright, 2021). Further, it is shown that yoga alleviates occupational stress in female nurses (Miyoshi, 2019). Yogic training improves physiological variables related to stress in working women (Manisha and Jayeshree, 2014).

## Discussion

It will be redundant to say that COVID-19 has drastically affected lives across the globe. Work from home and flexible working schedules are generally deemed to be more convenient ways of working. But during the global lockdown due to the pandemic, remote working poses a deterrent, especially to women. Women have to balance work and family duties, keep everyone in the household calm and safe, take care of the additional hygiene issues, keep up with childcare, and care of dependents without any external help. Women are facing gender disparity both at work and home more during the pandemic. This brings her at risk of being over-stressed and faces burnout. A study has shown that work-life conflict in women affected mental health adversely through negative affect and perceived stress. Besides, work-life conflict leads to increase in cortisol level, change in HPA axis activity, and negative affect. It is established to be a potent risk factor for stress and depression (Zhou et al., 2018). Yoga is proven to be a potent tool for enhancing mental health and reducing stress (Jasti et al., 2020). Although these findings can be confounded by various factors, studies conducted by Streeter et al., demonstrated that there is yoga-specific modulation of HPA axis leading to parasympathetic dominance (Streeter et al., 2010). Experienced yoga practitioners and yoga-naïve subjects show similar impact of yoga in the same duration implying that yoga takes minimal time to have an impact in case of physiological variables (Shinba et al., 2020). Change in psychological aspect may take some time to take effect and it varies depending on the duration of exposure to yoga. Yoga has a beneficial effect on physical, mental, and psychological health by integration of bottom-up and top-down mechanisms affecting the Hypothalamic-pituitary-adrenal axis and autonomic nervous system. This further, implies that yoga can enhance resilience and mental balance (Sullivan et al., 2018). Work-life balance needs certain resources and support that yoga can help provide. Yoga can, thus, be a viable option to help women sail through the tide safely and achieve work-life balance gently and smoothly.

## Conclusion

Work-life balance is disturbed in women due to “public and private patriarchy” (Docka-Filipek and Stone, 2021). Their mental health is deteriorated and more so during the pandemic. So far, no studies have been conducted to assess the impact of yoga on work-life balance of working women. The opinion article tries to propound this theory that yoga can be a cost-effective and viable tool to improve work-life balance in working women. This speculation arises from the beneficial effect yoga has on mental health and resilience. However, a study with robust study design should be conducted to arrive at concrete evidence.

## Author Contributions

RR has contributed to the conception of the idea. RR and EO have written sections of the manuscript and contributed to manuscript revision, read and approved the submitted version.

## Conflict of Interest

The authors declare that the research was conducted in the absence of any commercial or financial relationships that could be construed as a potential conflict of interest.

## Publisher's Note

All claims expressed in this article are solely those of the authors and do not necessarily represent those of their affiliated organizations, or those of the publisher, the editors and the reviewers. Any product that may be evaluated in this article, or claim that may be made by its manufacturer, is not guaranteed or endorsed by the publisher.
